# Delayed surgical debridement in pediatric open fractures: a systematic review and meta-analysis

**DOI:** 10.1007/s11832-014-0567-2

**Published:** 2014-02-20

**Authors:** Talal Ibrahim, Muhammad Riaz, Abdelsalam Hegazy, Patricia J. Erwin, Imad M. Tleyjeh

**Affiliations:** 1 Department of Orthopedic Surgery, Hamad General Hospital, Weill Cornell Medical College in Qatar, P.O. Box 3050, Doha, Qatar; 2 Division of Population Health Sciences and Education, University of London, London, UK; 3 Mayo Medical Library, Mayo Clinic, Rochester, USA; 4 Department of Medicine, King Fahad Medical City, Riyadh, Saudi Arabia

**Keywords:** Open fracture, Children, Debridement, Meta-analysis, Systematic review

## Abstract

**Purpose:**

Open fractures are considered orthopedic emergencies that are traditionally treated with surgical debridement within 6 h of injury to prevent infection. However, this proclaimed “6-h rule” is arbitrary and not based on rigorous scientific evidence. The aim of our study was to systematically review the literature that compares late (>6 h from the time of injury) to early (<6 h from the time of injury) surgical debridement of pediatric open fractures.

**Methods:**

We searched several databases from 1946 to 2013 for any observational or experimental studies that evaluated late and early surgical debridement of pediatric open fractures. We performed a meta-analysis using a random effects model to pool odds ratios for a comparison of infection rates between children undergoing late versus early surgical debridement. We also investigated the infection rates in upper- and lower-limb pediatric open fractures. Descriptive, quantitative, and qualitative data were extracted.

**Results:**

Of the 12 articles identified, three studies (retrospective cohort studies) were eligible for the meta-analysis, encompassing a total of 714 open fractures. The pooled odds ratio (OR = 0.79) for infection between late and early surgical debridement was in favor of late surgical debridement but was not statistically significant (95 % CI 0.32, 1.99; *p* = 0.38, *I*^2^ = 0 %). No significant difference in infection rate was detected between pediatric open fractures in the upper and lower limbs according to the time threshold in the included studies (OR = 0.72, 95 % CI 0.29, 1.82; *p* = 0.40, *I*^2^ = 0 %).

**Conclusions:**

The cumulative evidence does not, at present, indicate an association between late surgical debridement and higher infection rates in pediatric open fractures. However, initial expedient surgical debridement of open fractures in children should always remain the rule. Thus, multi-center randomized controlled trials or prospective cohort studies will be able to answer this question with more certainty and a higher level of evidence.

**Level of evidence:**

Level III.

## Introduction

Open fractures are considered orthopedic emergencies that are traditionally treated with surgical debridement, fracture stabilization, and the administration of intravenous antibiotics and tetanus prophylaxis. The initial surgical debridement is usually performed within 6 h from the time of injury to reduce the risk of infection. However, this proclaimed “6-h rule” is not based on rigorous scientific evidence; it originated from a study conducted by Friedrich on guinea pigs in the pre-antibiotic era in 1898 [1]. Several studies performed since then have challenged this rule and reported that the timing of surgical debridement of open fractures may not play such a critical role in the prevention of infection [2–4], particularly since the introduction of antibiotics [5–7]. Despite the lack of scientific evidence, Gustilo and Anderson’s classic article concluded that open fractures require emergency treatment, including adequate debridement and copious irrigation. The study did not specifically assess the relationship between surgical delay and infection rate, and remains highly referenced in the orthopedic literature [8].

Patzakis and Wilkins reviewed more than 1,000 open fractures and concluded that “the single most important factor in reducing infection rate was the early administration of antibiotics.” In this study, patients who were administered antibiotics within 3 h of injury had an infection rate of 4.7 %, compared to 7.5 % in those whom antibiotic treatment was administered 3 h or more after injury [9]. In a Cochrane review, Gosselin et al. [10] demonstrated a significant reduction in wound infections in patients who received antibiotic prophylaxis for all types of open fractures when compared with patients who received no antibiotic prophylaxis. Despite the importance of antibiotic administration in open fractures, the exact length of treatment remains controversial and arbitrary. In the pediatric open fracture literature, most studies suggest that intravenous antibiotic treatment should be administered for at least 48 h [11–14].

More recently, several authors have questioned the need for surgical debridement of Gustilo and Anderson type I open fractures in pediatric patients. The risk of infection is correlated with the type of soft tissue wound associated with the open fracture, and the rationale for surgical debridement of an open fracture is to protect against infection by meticulously debriding all devitalized tissue and copiously irrigating the wound to decrease the bacterial load. While there is no debate over the need for surgical debridement of Gustilo and Anderson type II and III open fractures, the controversy over type I open fractures remains. Yang and Eisler [15], in a retrospective study of both adults and children with isolated type I open fractures, reported a 0 % infection rate. Several other case series report infection rates of 2.5–4.0 % with nonsurgical treatment of pediatric open fractures, and consider this to be safe compared to the infection rate of pediatric type I open fractures treated with surgical debridement [16, 17].

Schenker et al. [18] carried out a meta-analysis to investigate the association between time to surgical debridement of open fractures in adults and infection. Their review of 16 studies showed no association between late surgical debridement and higher infection rates when all infections, deep infections, and more severe open fractures were considered.

The aim of our study was to systematically review the literature that compares late (>6 h from the time of injury) to early (<6 h from the time of injury) surgical debridement of pediatric open fractures. The primary outcome analysis involved the rate of infection.

## Materials and methods

### Search strategy

A senior medical librarian with 40 years of experience developed the search strategy and performed the literature search. The databases that were searched included Ovid MEDLINE (1946–October 2013), Ovid EMBASE (1988–2013), Web of Science, Elsevier Scopus, and the Cochrane Registry of Clinical Trials. The primary terms were “open fracture(s)” combined with “wound infection” and “debridement.” Articles were limited to randomized controlled trials, prospective or retrospective cohort studies, and case–control studies of pediatric patients. Two authors independently assessed the eligibility of identified studies. The full text of any study that could be relevant based on the respective abstract was reviewed. Bibliographies and review articles were reviewed manually for additional citations. Publication language was restricted to English. We did not seek unpublished investigations.

### Study selection

We considered randomized controlled trials, prospective or retrospective cohort studies, and case–control studies that directly compared late (>6 h from the time of injury) with early (<6 h from the time of injury) surgical debridement of pediatric open fractures and reported the rate of infection. An open fracture was defined as a fracture with bone exposed to the environment and communicating with the skin. The delay in surgical debridement was classified into late surgical debridement (>6 h from the time of injury) or early surgical debridement (<6 h from the time of injury).

### Data collection

Two authors independently extracted and recorded the required datasets, which included study characteristics (i.e., country, year of study), mean age of children, number of open fractures, number of infections, type of open fracture according to the Gustilo and Anderson classification, and location of the open fracture. Two authors independently assessed the methodological quality of the selected studies according to key validity components that address selection, comparability, and exposure using the Newcastle–Ottawa Scale [19] to assess the quality of nonrandomized studies. Any disagreement was resolved by consensus.

### Statistical methods

We pooled studies and constructed Forest plots using the DerSimonian–Laird random effects model [20], which assumes that the studies are a sample of all potential studies, and incorporates a between-study random effect component to allow for between-study heterogeneity. Between-study heterogeneity was quantified using the *I*^2^ statistic. This defines the variability percentage in effect estimates that is due to heterogeneity rather than to chance: the larger the *I*^2^, the greater the heterogeneity.

We based the main meta-analytic comparison on the odds ratio of infection rates in children undergoing late (>6 h from the time of injury) versus those undergoing early (<6 h from the time of injury) surgical debridement. Infection rates were obtained by dividing the number of open fractures that developed an infection by the total number of open fractures sustained. If no event occurred in at least one cell of the (2 × 2) contingency table for a parent study, a continuity correction of 0.5 was added to each cell to compute odds ratio and permit analysis, as described in the Cochrane handbook [21].

The diagnosis of infection was defined by clinical findings, surgical debridement or antibiotic treatment. We also undertook a comparison of the overall rate of infection in upper- and lower-limb pediatric open fractures without considering late and early surgical debridement.

Additional sensitivity analyses were performed to determine the rate of infection according to the Gustilo and Anderson classification for studies that had provided this information. A further sensitivity analysis was conducted to determine whether imputed results for the six open fractures with no time to surgical debridement recorded in the Kreder and Armstrong [22] study would affect the results if these open fractures were included in either the late or early surgical debridement groups.

## Results

### Yield of the search strategy and eligible studies

**Fig. 1:**
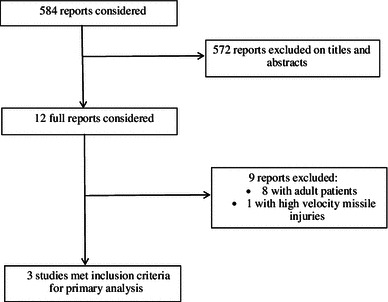
Flow diagram of eligible studies

The search strategy yielded 584 publications, among which we considered 12 articles for full-text review. We excluded nine studies as they did not fulfill our inclusion criteria for a comparison of late (>6 h from the time of injury) versus early (<6 h from the time of injury) surgical debridement of open fractures in children. A total of three studies addressing late versus early surgical debridement were eligible [22–24]. Figure 1 summarizes the process of identifying eligible studies. All three studies were retrospective cohort studies. There were no randomized controlled trials or prospective cohort studies. The kappa statistic for interobserver agreement on study eligibility was 1.0.

### Characteristics of the included studies

Table 1 summarizes the characteristics of the three studies included in our primary and sensitivity analyses. The studies included a total of 714 pediatric open fractures with a total of 26 infections. The individual sample sizes of the studies ranged from 50 to 554 open fractures. One of the studies was a multi-center trial including six tertiary pediatric centers. Kreder and Armstrong [22] only investigated open lower limb (tibia) fractures. All of the patients were children. The definition of infection was similar in all three studies. Only one study utilized positive intraoperative cultures as one of its criteria to define infection. The overall rate of infection for all of the studies included was 3.6 %. The majority of the pediatric open fractures were type I Gustilo–Anderson injuries that involved the lower limb. The time to surgical debridement in the comparison between the late and early surgical debridement groups was 6 h in all three studies included. All open fractures were followed up until both clinical and radiographic bone union.

**Table 1:** Characteristics of the studies included in the meta-analysis

Source, country	Mean age (range)	Number of open fractures	Type of open fracture^a^			Fracture location	Number of infections
			Type I	Type II	Type III		
Kreder and Armstrong [22], USA	10 (3–17)	56	14	16	26	Tibia	8
Skaggs et al. [23], USA	6.3 (0.9–17)	104	63	23	18	Upper limb	2
						Lower limb	
Skaggs et al. [24], USA (multi-center)	8.8 (0.2–18)	554	302	154	98	Upper limb	16
						Lower limb	
						Pelvis	

^a^According to the Gustilo–Anderson classification

### Quality assessment of the studies included

Table 2 summarizes the results for the different domains of study quality adapted from the Newcastle–Ottawa scale [19]. All three studies scored the maximum number of stars on the selection and outcome domains. None of the three studies specified the extent of the comparability of the late (>6 h from the time of injury) and early (<6 h from the time of injury) surgical debridement groups. All three studies scored a total of seven out of a maximum of nine stars. The kappa statistic for interobserver agreement on these quality domains was 1.0.

**Table 2:** Assessment of the quality of the studies included in the meta-analysis (Newcastle–Ottawa Scale)

Domain	Item	Kreder and Armstrong [22]	Skaggs et al. [23]	Skaggs et al. [24]
Selection (maximum of 4 stars)	Representativeness of the exposed cohort	*	*	*
	Selection of the unexposed cohort	*	*	*
	Ascertainment of exposure	*	*	*
	Demonstration that outcome of interest was not present at start of study	*	*	*
Comparability (maximum of 2 stars)	Comparability of cohorts on the basis of the design or analysis	Nil	Nil	Nil
Outcome (maximum of 3 stars)	Assessment of outcome	*	*	*
	Was follow-up long enough for outcome to occur?	*	*	*
	Adequacy of follow-up of cohorts	*	*	*

Maximum number of stars is 9 for the three domains

### Quantitative results of the meta-analysis

**Fig. 2:**
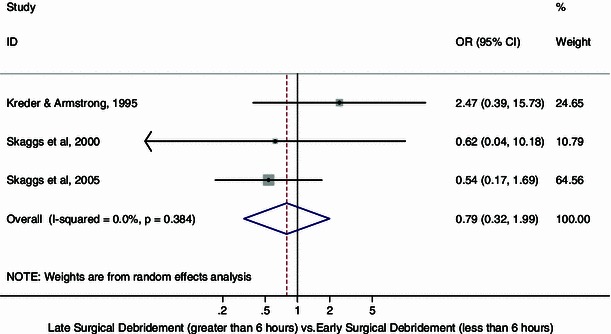
Forest plot: pooled odds ratio for infection in the late versus early surgical debridement groups

Figure 2 displays the cumulative meta-analytic comparison. A random-effects model meta-analysis of the three studies resulted in an overall pooled odds ratio of infection of 0.79 (95 % CI 0.32, 1.99; *p* = 0.38, *I*^2^ = 0 %) which suggested a lower infection rate in children who had late (>6 h from the time of injury) surgical debridement, but this difference was not statistically significant. The rates of infection in the late and early surgical debridement groups were 2.5 and 4.2 %, respectively.

**Fig. 3:**
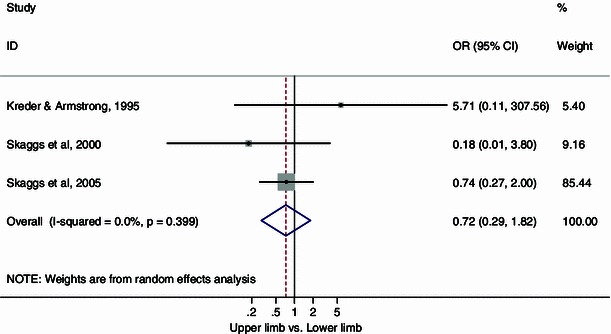
Forest plot: pooled odds ratio for infection in those with upper versus lower limb open fractures

Analysis of the overall pooled odds ratio for infection in those with upper versus lower limb pediatric open fractures, regardless of the time to surgical debridement, was in favor of upper limb fractures, but this difference did not reach statistical significance (OR = 0.72, 95 % CI 0.29, 1.82; *p* = 0.40, *I*^2^ = 0 %) (Fig. 3). The rates of infection in those with open upper and lower limb fractures were 2.1 and 5.1 %, respectively.

Because of the limited number of studies, we did not undertake statistical analysis of funnel plots to assess publication bias [25].

**Fig. 4:**
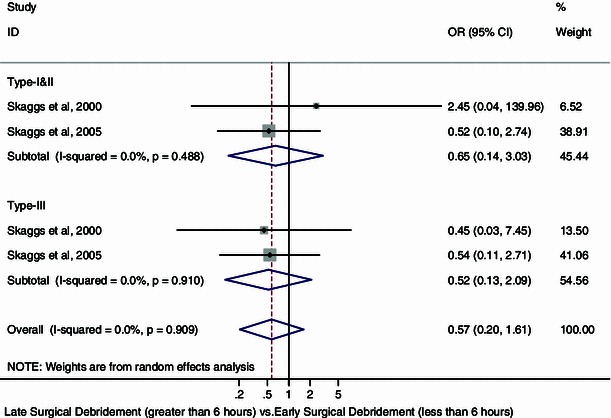
Forest plot: pooled odds ratio for infection according to the Gustilo and Anderson open fracture classification

Sensitivity analysis, with the exception of the study by Kreder and Armstrong [22] (due to a lack of information regarding the type of Gustilo and Anderson open fractures present in those who suffered infections), revealed pooled odds ratios for infection of 0.65 (95 % CI 0.14, 3.03; *I*^2^ = 0 %; *p* = 0.49) for type I and II Gustilo and Anderson open fractures and 0.52 (95 % CI 0.13, 2.09; *I*^2^ = 0 %; *p* = 0.91) for type III Gustilo and Anderson open fractures, respectively, in favor of late surgical debridement (Fig. 4). However, the differences were not significant.

Sensitivity analysis using imputed results for the six open fractures reported in the Kreder and Armstrong study [22] with no recorded times to surgical debridement revealed only minimal changes in the odds ratios and no significant changes in the overall results.

## Discussion

Late surgical debridement was associated with a pooled rate of infection of 2.5 %, which was not higher than the infection rate of 4.2 % rate seen for early surgical debridement in children with open fractures. Our study also found that the rate of infection in open upper limb fractures was not different from that for open lower limb fractures in children. Consequently, in certain circumstances,orthopedic surgeons may have to delay the surgical debridement of open fractures in children to optimize their condition, and our study confirms that there is no harm in delaying surgical debridement. Our findings are consistent with the literature on adult open fractures, in that late surgical debridement was not associated with higher infection rates and that the “6-h rule” has little support in the available literature [18]. The effect of late surgical debridement on treatment is likely to be small, and reproducing the current study designs with greater power may only serve to render a clinically insignificant difference statistically significant. The results were consistent across different assumptions. The extent to which this statement reflects the true outcome of the comparison requires an understanding of the limitations of the current literature and the included studies and a consideration of how the analyses were conducted and interpreted. The ability to detect a difference is further confounded by the relatively small sample size. Although all of the included studies assessed the effect of delayed surgical debridement in children, there was variation in the reporting of the key determinants of pediatric open fractures that are known to influence the infection rate, and none of these studies reported effect estimates that had been adjusted for these potential confounders.

Our systematic review identified three retrospective cohort studies [22–24] (level III) that compared the rate of infection in children who underwent late surgical debridement with the corresponding rate in those who underwent early surgical debridement. All studies were of good methodological quality according to the Newcastle–Ottawa scale, with limitations in the comparability domain. Because of the small number of studies included, we did not incorporate quality into our sensitivity analysis. The simplest approach is to judge each study based on specific domains of quality that are most relevant to the control of bias for that particular study.

A limitation of our analysis is the paucity of studies that address this pivotal issue. Only three published studies were eligible, but we chose to perform the meta-analysis to provide more generalizable results on the effect estimate. The only outcome measure examined in this meta-analysis was the rate of infection. This is a clinically relevant and important outcome, and the three studies had a similar definition of infection. Two of the studies categorized their infections as either superficial or deep. Other important factors, such as the type and time of antibiotic administration, type and amount of wound debridement, irrigation practices, method of wound closure, type of fracture fixation, patient co-morbidities, injury characteristics, skeletal instability, and more importantly the accuracy of the time recorded between the injury and the abovementioned variables could not be controlled for in this analysis and require further study. Given the limited number of studies that address these factors, it is only possible to draw limited conclusions from the current study. These factors will vary in particular from center to center and are more relevant in the multi-center study reported by Skaggs et al. [24]. The same multi-center study reported by Skaggs et al. [24] also considered both superficial and deep infections collectively. We were unable to investigate the effect of the depth of infection using subgroup analysis because of the lack of data available in the studies. The study reported by Kreder and Armstrong [22] did not include the delay times to surgical debridement for six open tibia fractures, and that study only consisted of pediatric open tibia fractures. Two of the included studies were reported by Skaggs et al. [23, 24], and some of the open fractures may have been duplicated because of the timeframe of the retrospective chart reviews for these studies. Publication bias is also possible in our meta-analysis. The small number of studies limits our ability to assess for (using a funnel plot) or draw conclusions regarding publication bias.

Our study only assessed the effect of time to surgical debridement on the rate of infection following open fractures, even though this is one of many factors that may influence infection. Consequently, orthopedic surgeons need not abide by the “6-h rule,” as this study has showed that there is no harm in delaying surgical debridement from 7 to 24 h following injury, but initial expedient surgical debridement of open fractures in children should always remain the rule. The results of our meta-analysis are based on observational studies, and further attention should be directed toward studies of good methodological quality with adequate follow-up. Therefore, multi-center randomized controlled trials or prospective cohort studies will be able to answer this question with more certainty and a higher level of evidence.
